# Supplementary material for: Mechanisms governing the pioneering and redistribution capabilities of the non-classical pioneer PU.1
Source: Nat Commun. 2020 Jan 21;11:402. doi: 10.1038/s41467-019-13960-2 (PMC6972792; doi:10.1038/s41467-019-13960-2)
Supplement: Supplementary file 7 — Source data [file 41467_2019_13960_MOESM7_ESM.zip › Source_Data/FigureS2/FigureS2B_MotifScanOutput/KmeansCluster8/homerResults/motif30.similar.html]

motif30

## Information for motif30

C
A
T
G
T
C
G
A
A
C
G
T
C
A
T
G
C
G
T
A
A
T
G
C
A
C
G
T
G
T
A
C
C
T
G
A
  
Reverse Opposite:  

G
A
C
T
C
A
T
G
G
T
C
A
T
A
C
G
C
G
A
T
G
T
A
C
C
G
T
A
A
G
C
T
G
T
A
C
  

|  |  |
| --- | --- |
| p-value: | 1e-19 |
| log p-value: | -4.377e+01 |
| Information Content per bp: | 1.668 |
| Number of Target Sequences with motif | 241.0 |
| Percentage of Target Sequences with motif | 15.94% |
| Number of Background Sequences with motif | 3896.3 |
| Percentage of Background Sequences with motif | 8.58% |
| Average Position of motif in Targets | 106.0 +/- 57.1bp |
| Average Position of motif in Background | 103.0 +/- 69.3bp |
| Strand Bias (log2 ratio + to - strand density) | 0.0 |
| Multiplicity (# of sites on avg that occur together) | 1.06 |
| Motif File: | file (matrix) reverse opposite |

### Similar de novo motifs found

|  |  |  |  |  |  |  |  |
| --- | --- | --- | --- | --- | --- | --- | --- |
| Rank | Match Score | Redundant Motif | P-value | log P-value | % of Targets | % of Background | Motif file |
| 1 | 0.954 | G T C A A C G T A C T G G T C A T A C G C G A T G T A C C G T A | 1e-17 | -40.454245 | 14.42% | 7.69% | motif file (matrix) |
| 2 | 0.821 | C A T G G T A C G C A T A C T G C T G A T A G C G C A T T G A C C G T A A G C T G T A C | 1e-16 | -36.934432 | 4.70% | 1.42% | motif file (matrix) |
| 3 | 0.822 | C A G T T G A C C G A T T C A G G T C A T C A G G A C T G A T C T C G A G C A T | 1e-14 | -32.411501 | 5.56% | 2.07% | motif file (matrix) |
| 4 | 0.791 | C A T G T G A C C G A T A T C G G C T A A G T C G C A T T G A C T G C A G A C T A G T C C G T A | 1e-13 | -31.564050 | 3.24% | 0.83% | motif file (matrix) |
